# Revitalizing Soybean Plants in Saline, Cd-Polluted Soil Using Si-NPs, Biochar, and PGPR

**DOI:** 10.3390/plants13243550

**Published:** 2024-12-19

**Authors:** Khadiga Alharbi, Emad M. Hafez, Nevien Elhawat, Alaa El-Dein Omara, Emadelden Rashwan, Hossam H. Mohamed, Tarek Alshaal, Samir I. Gadow

**Affiliations:** 1 Department of Biology, College of Science, Princess Nourah bint Abdulrahman University, P.O. Box 84428, Riyadh 11671, Saudi Arabia; kralharbi@pnu.edu.sa; 2 Department of Agronomy, Faculty of Agriculture, Kafrelsheikh University, Kafr El-Sheikh 33516, Egypt; 3 Institute of Agricultural Resources and Environment, Jiangsu Academy of Agricultural Sciences, Nanjing 210014, China; 4 Department of Applied Plant Biology, Faculty of Agricultural and Food Sciences and Environmental Management, University of Debrecen, Böszörményi Str. 138, 4032 Debrecen, Hungary; 5 Faculty of Agriculture (for Girls), Al-Azhar University, Tanta 31732, Egypt; 6 Department of Microbiology, Soils, Water Environment Research Institute, Agricultural Research Center, Giza 12112, Egypt; alaa.omara@yahoo.com; 7 Agronomy Department, Faculty of Agriculture, Tanta University, Tanta 31527, Egypt; emad.rashwan@agr.tanta.edu.eg; 8 Agronomy Department, Faculty of Agriculture, Ain Shams University, Cairo 11566, Egypt; dr.hossam16@yahoo.com; 9 Soil and Water Department, Faculty of Agriculture, Kafrelsheikh University, Kafr El-Sheikh 33516, Egypt; 10 Department of Agricultural Microbiology, Agriculture and Biology Research Institute, National Research Centre, 33 EI Buhouth St., Dokki, Cairo 12622, Egypt; si.gadow@nrc.sci.eg

**Keywords:** Cd-contaminated soil, soil salinity, soil enzymes, microbial activity, soil remediation, plant nutrition

## Abstract

Excessive irrigation of saline-alkaline soils with Cd-contaminated wastewater has resulted in deterioration of both soil and plant quality. To an investigate this, a study was conducted to explore the effects of biochar (applied at 10 t ha^−1^), PGPRs (*Bradyrhizobium japonicum* (USDA 110) + *Trichoderma harzianum* at 1:1 ratio), and Si-NPs (25 mg L^−1^) on soybean plants grown in saline-alkali soil irrigated with wastewater. The results showed that the trio-combination of biochar with PGPRs, (as soil amendments) and Si-NPs (as foliar spraying), was more effective than individual or coupled applications in reducing Cd bioavailability in the soil, minimizing its absorption, translocation and bioconcentration in soybean tissues. The trio-combination reduced Cd bioavailability in the soil by 39.1% and Cd accumulation in plant roots, shoots, and seeds by 61.0%, 69.3%, and 61.1%, respectively. Physiological improvements in soybean plants were also observed, including 197.8% increase in root growth, 209.3% increase in chlorophyll content, and 297.4% increase in carotenoid levels. The trio-combination significantly improved soil physicochemical characteristics, enhanced soil microbial indicators and boosted soil enzymes activity, which in turn facilitated nutrient uptake and increased antioxidant enzymes activity. These positive outcomes enhanced photosynthesis, improved productivity and increased seed nutritional value. Overall, the trio-combination of biochar with PGPRs and Si-NPs are considered a reliable approach not only for revitalizing soybean growth but also for immobilizing Cd and improving soil health under wastewater irrigation.

## 1. Introduction

Soybean (*Glycine max* L.) is widely recognized as oilseed crop, providing more than 35% of the world’s oil and 70% of its protein for food and feed, with an annual production of approximately 350 million tons [1]. In addition to its economic significance, soybean plays a crucial role in enhancing soil health through biological nitrogen fixation. Beyond its contributions to food and feed, soybean is also a valuable source of nutraceutical compounds, bioenergy, and has various medical benefits [2]. Given these numerous advantages, soybean deserves more attention than it currently receives. However, soybean is somewhat sensitive to salt and faces significant challenges due to various environmental constraints, particularly when cultivated on soils contaminated with heavy metals or subjected to salt stress [3]. Despite its multifaceted utilities, soybean cultivation faces considerable threats, especially from irrigation with wastewater polluted with heavy metals such as cadmium (Cd). This contamination not only affects soil health but also poses severe risks to the safety and productivity of the soybean crop when grown in salt-affected soils [4,5].

Reliance on agricultural and industrial wastewater for irrigation presents significant challenges and hazards, which must be wisely managed [1]. Freshwater resources have become extremely scarce as a result of overuse, and as a result, drainage water—that is often easily accessible in agricultural regions, has emerged as a primary source of irrigation water [6,7]. In Egypt, the Kitchener Drain, a major source of heavy metal pollution, irrigates 191,213 hectares annually [8,9]. Among the various contaminants in drainage water, cadmium (Cd) is considered the most prominent heavy metal, primarilyoriginating fromindustrial activities [10]. So, drainage water contaminated with Cd poses severe risks to soil, crops, animals, and human health. Cd stress induces metabolic disruptions, tissue necrosis and restrains nutrient uptake, leading to oxidative damage [11,12]. Soil contamination with Cd negatively impacts its physiochemical properties, adversely physiological functions and structural changes including root growth inhibition, decreased leaf relative water content, decreased stomatal conductance, and impaired enzymes activity [13]. Cd is particularly hazardous because of its rapid absorption by plant roots and its fast accumulation in various plant tissues, potentially causing direct toxicity even at low soil concentrations [14]. Irrigation with Cd-polluted wastewater, a common contaminant of farmland soil, can lead to significant accumulation in plant tissues, resulting in stunted growth and severe yield reductions [15]. Therefore, mitigating Cd pollution in soils and reducing its bioavailability has become a critical concern [16].

Soil exposure to salinity, at varying levels, is one of the most detrimental conditions for Soybean crops [17]. The negative effects of salinity begin at the seed germination stage, during nodulation, and root growth, and seed formation due to osmotic damage and ionic imbalance [18]. Soybean is considered a moderately salt-sensitive crop, with potential yield reductions 40% due to salinity [19]. Salt stress disrupts Soybean metabolism and induces oxidative damage; also protectants, beneficial microbes, or proper management strategies can help boost tolerance [20].

Plant growth-promoting rhizomicrobes (PGPR) improve soil quality, root growth, and overall plant health in heavy metal-contaminated or salt-affected soils, enhancing crop yield and nutrient uptake by mitigating abiotic stress and pollutants [21]. The major importance of PGPR in this context lies in their potential to Cd detoxify and combat salt stress in the soil. They achieve this through mechanisms such as reducing soil pH, inducing phytohormones, lowering ABA content, maintaining ionic balance and scavenging ROS, all of which contribute to better root growth and nutrient uptake [22]. PGPR application could help maintain N fixation, which enhances soil physicochemical characteristics and improves Cd-detoxification via processes like reduction, oxidation, and methylation or demethylation collectively known as phytoremediation. This process results in the immobilization, chelation, and active removal of Cd due to increased auxin production [23]. PGPR strains, such as *Bradyrhizobium japonicum* (USDA 110) and *Trichoderma harzianum* are well-fitted to soils contaminated with heavy metal and affected by salinity, as they form a symbiotic relationship with soybean, fixing nitrogen efficiently [24]. Co-inoculation of Soybean with these PGPR strains can significantly improve soil enzymes activity, nodulation, root growth, ionic equilibrium and nutrient uptake, leading to enhanced biosynthesis of metabolites, better physiological performance and increased seed yield [17,18]. Thus, PGPR have significant potential to alleviate the effects of salt stress while promoting Cd-detoxification in contaminated soils [14]

Biochar is an organic passivation material, carbon-rich with a high surface area and internal porosity, produced through the pyrolysis of plant residues in a muffle furnace under hypoxic conditions at 450 °C for 3 h [14,25]. Biochar play a cruical role in detoxifying Cd, immobilizing soil Cd, and enhancing nutrient availability, microbial activity, and biosynthesis in salt-affected soils [26]. It significantly improves soil traits by augmenting soil respiration, reducing soil pH, that helps maintain ionic and osmotic equilibrium [27]. As a soil amendment, biochar offers a multifaceted approach to reduce Cd-toxicity by promoting lower Na^+^ and higher K^+^ accumulation. It also improves plant hormone synthesis, decreases ROS generation and enhances antioxidant enzymes activity, all of which contribute to improved growth, development, and yield [28].

Efforts to reduce the uptake, transport, and accumulation of Cd and sodium ions from soil to plants are critical for ensuring food safety [17,29]. Silicon nanoparticles (Si-NPs) help prevent Cd and Na accumulation in plants due to their small size and high flexibility [30]. The use of Si-NPs offers a promising strategy, as they are easily absorbed by plants, improving nutrient uptake, enhancing crop quality, and contributing to environmental sustainability [31]. Foliar spraying with Si-NPs offers several advantages, including biocompatibility, biodegradability and non-toxicity. Additionally, Si-NPs helpincrease resistance to oxidative stress, improve photosynthesis, boost crop yield and enhance nutrient uptake in edible crops [32]. Silicon (Si), the second richest element in the earth’s crust, plays a key role in regulating plant bioprocesses under ecological stresses such as heavy metal accumulation and salt stress [33]. Most of silicon absorped by plants helps inhibit the uptake, translocation and accumulation of Cd and Na, as described in various workers [34,35]. The appropriate use of eco-friendly Si-NPs is particularly effective in immobilizing Cd and Na as shown in different crops such as, rice [36], maize [2,37], bean [38,39] and soybean [17,40]. As a highly effective adsorbent, Si-NPs have vast agricultural and biotechnological applications. They help combat Cd and Na ions in plants, reducing oxidative stress, scavenging ROS and promoting biochemical processes and morphological improvements [41].

This study aims to evaluate the integrated effects of biochar, plant growth-promoting rhizomicrobes (PGPR), and silicon nanoparticles (Si-NPs) in mitigating cadmium (Cd) stress in soybean cultivated in saline-alkali soils irrigated with wastewater. The investigation focuses on assessing the influence of these treatments on soil chemical properties, biological activity, enzymatic activity, and the uptake of Cd and sodium (Na) ions in different plant tissues, including roots, shoots, and seeds. Furthermore, the study examines oxidative stress markers, antioxidant enzyme activities, gas exchange parameters, physiological functions, and ion homeostasis. The overarching objective is to determine how these combined applications enhance yield traits and improve the nutritional quality of soybean seeds.

## 2. Results

### 2.1. Changes in Soil Biochemical and Enzymes Activity in Response to Biochar, PGPR and SiNPs

**Figure 1:**
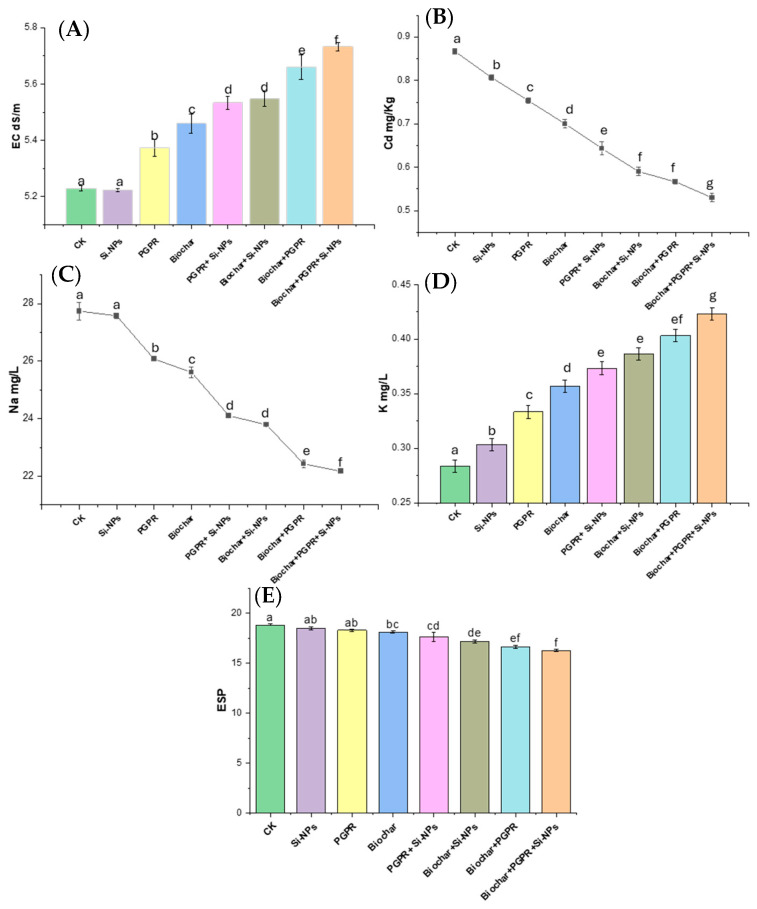
(**A**) EC (dS m^−1^), (**B**) soil Cd content (mg kg^−1^), (**C**) Na content (mg L^−1^), (**D**) K content (mg L^−1^) and (**E**) ESP, in soil grown with soybean (*Glycine max* L.), irrigated with wastewater Cd-polluted and treated with single, coupled and trio-combination of biochar at rate of 10 t ha^−1^, PGPR at rate of (1:1) and Si-NPs (25 mg L^−1^) compared to untreated plants (control; CK). Different letters on bars are significant based on the Tukey’s HSD test (*p* < 0.05).

**Figure 2:**
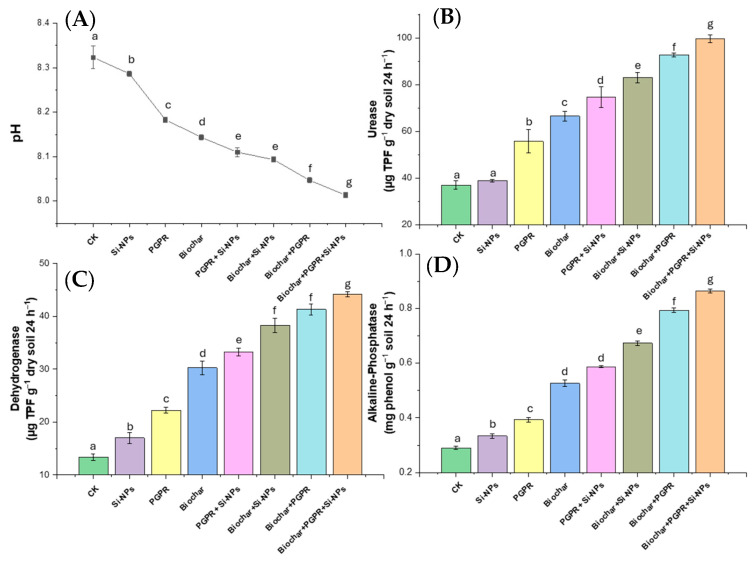
(**A**) pH, (**B**) urease (µg TPF g^−1^ dry soil 24 h^−1^), (**C**) dehydrogenase (µg TPF g^−1^ dry soil 24 h^−1^) and (**D**) alkaline-phosphatase (mg phenol g^−1^ dry soil 24 h^−1^) in soil grown with soybean (*Glycine max* L.), irrigated with wastewater Cd-polluted and treated with single, coupled and trio-combination of biochar at rate of 10 t ha^−1^, PGPR at rate of (1:1) and Si-NPs (25 mg L^−1^) compared to untreated plants (control; CK). Different letters on bars are significant based on the Tukey’s HSD test (*p* < 0.05).

Figure 1 and Figure 2 illustrate the effects of various treatments on the physicochemical properties and enzyme activities of soils planted with soybean. Notably, the trio-combination of biochar, PGPR, and Si-NPs demonstrated the most significant improvements across most parameters, highlighting a synergistic interaction among these components that enhances soil health and functionality.

All treatments resulted in a decrease in pH compared to the CK, with the most pronounced reduction, 3.7%, occurring in the trio combination of biochar, PGPR, and Si-NPs. In contrast, electrical conductivity (EC) showed an increasing trend across all treatments, with the highest EC increase (9.56%) also observed in the trio combination. Cadmium levels were significantly reduced, dropping by 39.1% under the same treatment. Likewise, sodium (Na) concentrations decreased across all treatments, with the trio combination showing the largest reduction at 63.16%. Potassium (K) levels, however, increased with all treatments except for Si-NPs, which showed a slight decrease. The highest K increase, 42.86%, was again noted in the trio combination. Finally, exchangeable sodium percentage (ESP) values decreased across all treatments, with the most substantial reduction of 3.24% seen in the trio combination.

Soil enzyme activities, such as urease, increased significantly with all treatments, with the highest rise (166.92%) observed in the trio combination of biochar, PGPR, and Si-NPs. Dehydrogenase activity also showed an increase across all treatments, peaking at 229.04% in the trio combination. Among the enzyme activities, alkaline phosphatase exhibited the most notable increases, with substantial gains seen across all treatments. The highest alkaline phosphatase activity, a 204.83% increase, was observed in the trio combination treatment. Overall, the findings demonstrate that all treatments, whether applied individually or as a trio combination, affected soil properties and enzyme activities in different ways. While pH decreased with all treatments, EC, Na, and K concentrations increased. ESP values declined, and all enzyme activities (urease, dehydrogenase, and alkaline phosphatase) showed significant improvements.

### 2.2. Changes in Soil CO_2_ Evolution and Soil Microbial Biomass Carbon in Response to Biochar, PGPR and SiNPs

**Figure 3:**
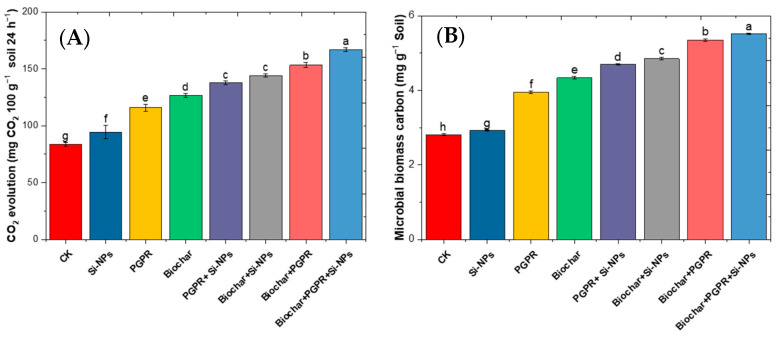
(**A**) soil CO_2_ evolution (mg CO_2_ 100 g^−1^ soil 24 h^−1^) and (**B**) microbial biomass carbon (SMBc; mg g^−1^ Soil) in soil grown with soybean (*Glycine max* L.), irrigated with wastewater Cd-polluted and treated with single, coupled and trio-combination of biochar at rate of 10 t ha^−1^, PGPR at rate of (1:1) and Si-NPs (25 mg L^−1^) compared to untreated plants (control; CK). Different letters on bars are significant based on the Tukey’s HSD test (*p* < 0.05).

Figure 3 highlights the effects of single, paired, and trio combinations of biochar, PGPR, and Si-NPs on soybean plants grown in saline-alkali soil irrigated with Cd-contaminated wastewater. Various treatments—including CK, biochar (10 t ha^−1^), PGPR (1:1), Si-NPs (25 mg L^−1^), and their trio-combination—were evaluated for their impact on soil microbial biomass, expressed as percentage changes relative to the CK. In the CK, CO_2_ evolution was 85.05 mg CO_2_ per 100 g of soil per 24 h, and microbial biomass carbon was 2.83 mg per g of soil. The Si-NPs treatment caused a modest increase, raising CO_2_ evolution by 3.87% and microbial biomass carbon by 3.88%. PGPR had a more pronounced effect, boosting CO_2_ evolution by 40.35% and microbial biomass carbon by 40.28%. Biochar alone was even more effective, increasing CO_2_ evolution by 51.46% and microbial biomass carbon by 52.30%. The combination treatments showed even stronger effects. PGPR and Si-NPs together increased CO_2_ evolution by 60.34% and microbial biomass carbon by 65.72%, while biochar and Si-NPs increased CO_2_ evolution by 70.61% and microbial biomass carbon by 72.45%. The combination of biochar and PGPR was particularly effective, raising CO_2_ evolution by 80.30% and microbial biomass carbon by 87.61%. The most significant effects were observed with the trio combination of biochar, PGPR, and Si-NPs, which resulted in a 94.42% increase in CO_2_ evolution and a 94.68% increase in microbial biomass carbon.

The observed increases in CO_2_ evolution and microbial biomass carbon are closely linked to the activity of soil enzymes. Soil enzymes are critical mediators of OM decomposition, processes that directly influence microbial activity and CO_2_ production. Treatments that enhance microbial biomass, such as the application of PGPR and biochar, likely stimulate enzyme activity by providing a more favorable environment for microbial proliferation. This, in turn, accelerates the breakdown of organic matter, leading to higher CO_2_ evolution rates. The synergy observed in trio-combination of treatments can be attributed to the complementary effects of different amendments on soil enzyme dynamics. For example, biochar can enhance enzyme stability and substrate availability, while PGPR can directly increase enzyme production through microbial growth promotion. Silicon nanoparticles may also contribute by improving plant resistance to stress, indirectly promoting microbial activity and enzyme function. Therefore, the significant enhancements in CO_2_ evolution and microbial biomass carbon observed in the trio-combination highlight the vital role of soil enzymes in motivating these processes.

### 2.3. Changes in Cadmium (Cd) Accumulation and Its Translocation in Different Plant Parts in Response to Biochar, PGPR and SiNPs

**Figure 4:**
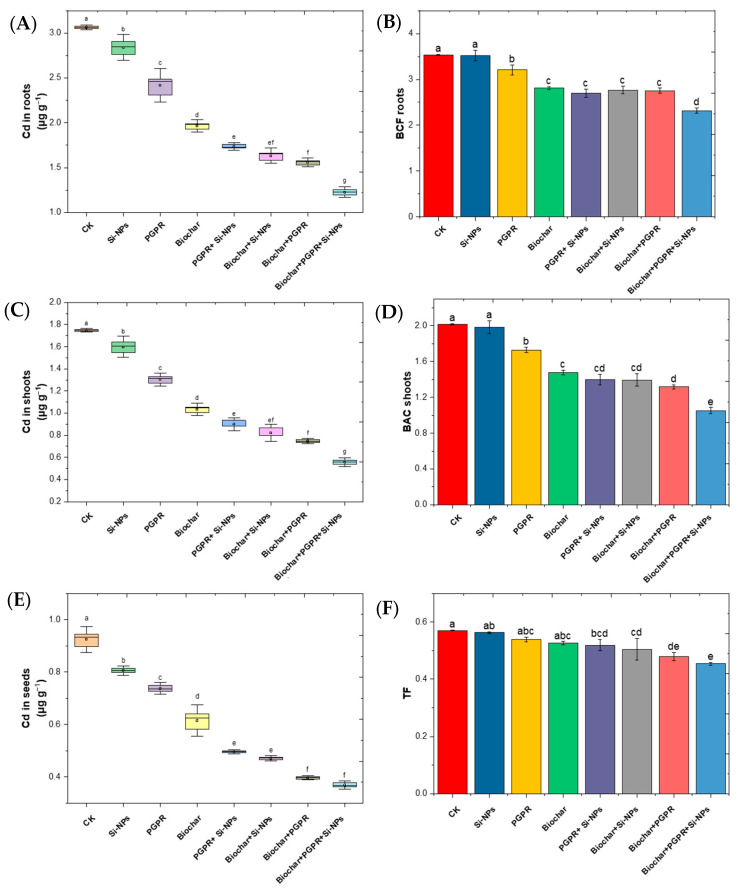
(**A**) Cd content in roots (µg g^−1^), (**B**) Cd content in shoots (µg g^−1^), (**C**) Cd content in seeds (µg g^−1^), (**D**) Bioconcentration factor (BCF), (**E**) bioaccumulation coefficient (BAC) and (**F**) translocation factor (TF) in soybean (*Glycine max* L.), irrigated with wastewater Cd-polluted and treated with single, coupled and trio-combination of biochar at rate of 10 t ha^−1^, PGPR at rate of (1:1) and Si-NPs (25 mg L^−1^) compared to untreated plants (control; CK). Different letters on bars are significant based on the Tukey’s HSD test (*p* < 0.05).

The results presented in Figure 4 evaluate the effects of single, combined, and trio applications of biochar, PGPR, and Si-NPs on soybean plants grown in saline-alkali soils irrigated with Cd-contaminated wastewater. A comparison of cadmium (Cd) accumulation in plant tissues and related parameters with the control (CK) reveals several notable changes. Specifically, all treatments significantly reduced Cd accumulation in roots compared to the control, demonstrating their efficacy in mitigating Cd uptake under stress conditions. The Si-NPs treatment reduced Cd accumulation by 9.8%, PGPR by 18.9%, Biochar by 37.1%, coupled PGPR and Si-NPs by 42.6%, coupled biochar and Si-NPs by 45.7%, coupled Biochar and PGPR by 50.0%, and trio-combination of biochar, PGPR and Si-NPs by 61.0%. This indicates that the combined treatments, particularly trio-combination of biochar, PGPR and Si-NPs, were most effective in reducing Cd uptake in roots. In shoots, all treatments also decreased Cd concentration significantly. Si-NPs led to a reduction of 11.4%, PGPR to 24.0%, biochar to 42.6%, coupled PGPR and Si-NPs to 49.5%, coupled biochar and Si-NPs to 54.2%, coupled biochar and PGPR to 56.5%, and trio-combination of Biochar, PGPR and Si-NPs to 69.3%. These results further underscore the efficacy of the combined treatments in mitigating Cd accumulation. For seeds, the reduction in Cd accumulation was also notable across all treatments. Si-NPs reduced Cd levels by 12.6%, PGPR by 19.6%, biochar by 33.0%, coupled PGPR and Si-NPs by 46.4%, coupled biochar and Si-NPs by 49.1%, coupled biochar and PGPR by 57.9%, and trio-combination of Biochar, PGPR and Si-NPs by 61.1%. Again, the combined treatments demonstrated the highest reduction, indicating their potential for minimizing Cd transfer to edible plant parts. The Bioconcentration Factor (BCF) in roots, which represents the ratio of Cd concentration in roots to that in soil, showed a decrease for all treatments. Si-NPs reduced BCF by 3.1%, PGPR by 7.1%, biochar by 20.5%, coupled PGPR and Si-NPs by 20.5%, coupled biochar and Si-NPs by 21.3%, biochar and PGPR by 23.6%, and trio-combination of biochar, PGPR and Si-NPs by 35.8%. These results suggest that the treatments not only reduce Cd uptake but also lower its relative concentration in roots compared to soil, with combined treatments being the most effective.

The Translocation factor (TF), that points out to efficiency of Cd transfer from roots to shoots, was mostly unaffected by the treatments, with only minor reductions observed. Si-NPs reduced TF by 1.8%, PGPR by 7.0%, Biochar by 8.8%, coupled PGPR and Si-NPs by 12.3%, coupled biochar and Si-NPs by 15.8%, coupled biochar and PGPR by 12.3%, and trio-combination of biochar, PGPR and Si-NPs by 21.1%. Although the changes in TF were less pronounced, the slight decreases suggest that these treatments can moderately impede Cd movement within the plant. The bioaccumulation coefficient (BAC) in shoots, reflecting the Cd accumulation in shoots relative to soil, followed trends similar to those observed for Cd concentration in shoots. Si-NPs reduced BAC by 5.0%, PGPR by 12.9%, Biochar by 27.9%, coupled PGPR and Si-NPs by 30.3%, coupled biochar and Si-NPs by 33.8%, coupled biochar and PGPR by 33.8%, and trio-combination of biochar, PGPR and Si-NPs by 49.8%. This indicates that the combined treatments, especially trio-combination of biochar, PGPR and Si-NPs, were most effective in reducing Cd bioaccumulation.

The combined application of biochar, PGPR, and Si-NPs consistently exhibited the highest efficacy in reducing cadmium (Cd) accumulation across all plant tissues and associated parameters, suggesting that this trio of treatments effectively mitigates Cd toxicity in plants. These treatments significantly decreased Cd uptake in roots, shoots, and seeds while also reducing the bioconcentration factor (BCF), translocation factor (TF), and bioaccumulation coefficient (BAC), highlighting their potential as a robust strategy for minimizing Cd bioavailability and toxicity in plants. The substantial reduction in Cd accumulation in these plant parts, compared to the CK, indicates that this combination could be particularly valuable for phytoremediation efforts or agricultural practices where soil Cd contamination threatens food safety and crop health. By decreasing the BCF, these treatments reduce Cd accumulation in the roots, the primary site of metal uptake from the soil. This reduction in root Cd content subsequently limits its translocation to the shoots and seeds, thereby minimizing overall Cd levels in the plant. Although the TF was not significantly altered, the absolute reductions in Cd content across the plant tissues are noteworthy. This suggests that while Cd movement from roots to shoots may not be heavily impeded, the overall availability and uptake of Cd are significantly lowered. The decrease in BAC in shoots further supports this, indicating lower Cd accumulation relative to soil content, and thus underscores the effectiveness of the treatments in reducing the risk of Cd entering the food chain through plant consumption.

The study clearly demonstrates that the use of biochar, PGPR, and Si-NPs, particularly in trio-combination of, offers a potent approach for mitigating Cd accumulation in plants. This trio-combination of treatment could serve as an effective strategy for addressing soil Cd contamination, thereby safeguarding crop health and reducing the risk of Cd exposure to humans and animals through the food chain. The findings underscore the importance of exploring integrated soil amendments and microbial treatments to enhance phytoremediation and ensure sustainable agricultural practices in contaminated environments.

### 2.4. Changes in Plant-Related Parameters Such as Root Growth and Chlorophyll Pigments in Soybean Plants in Response to Biochar, PGPR and SiNPs

**Figure 5:**
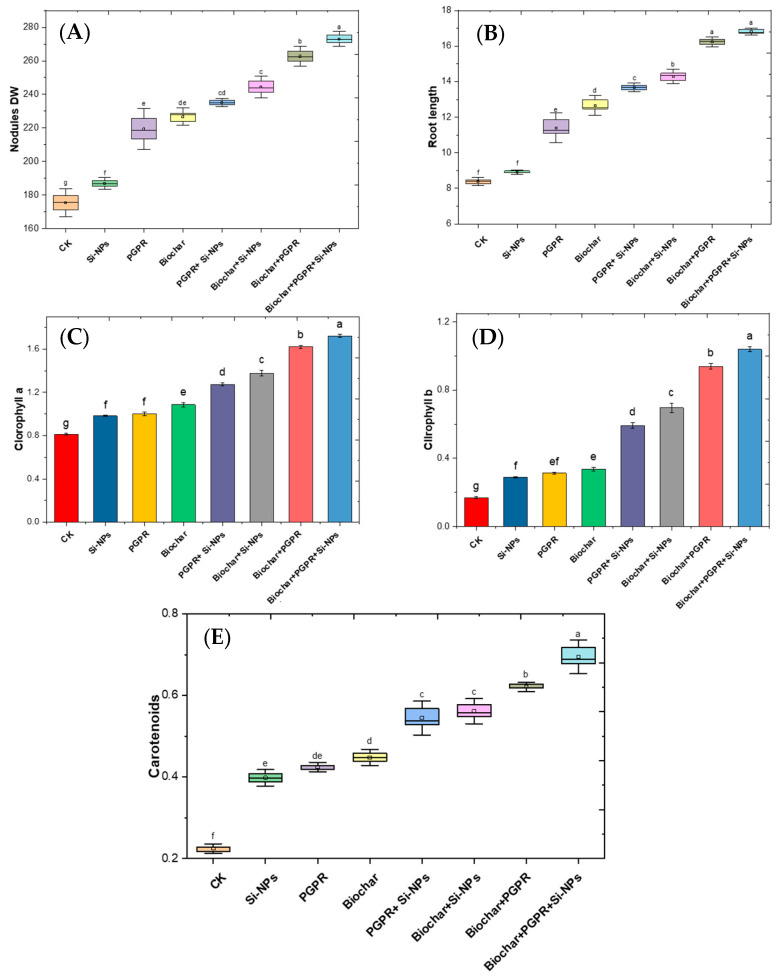
(**A**) nodules dry weight (mg plant^−1^), (**B**) root length (cm), (**C**) chlorophyll a (mg g^−1^ FW), (**D**) chlorophyll b (mg g^−1^ FW), and (**E**) carotenoids (mg g^−1^ FW) in soybean (*Glycine max* L.), irrigated with wastewater Cd-polluted and treated with single, coupled and trio-combination of biochar at rate of 10 t ha^−1^, PGPR at rate of (1:1) and Si-NPs (25 mg L^−1^) compared to untreated plants (control; CK). Different letters on bars are significant based on the Tukey’s HSD test (*p* < 0.05).

The results in Figure 5 illustrate the impacts of single, coupled and trio-combination of application biochar, PGPR and SiNPs on soybean plants subjected to saline-alkali soil irrigated with Cd-contaminated wastewater. Figure 5 illustrates the impact of biochar, PGPR, and Si-NPs, and their trio-combination on nodules dry weight (DW), root length, chl a, chl b, and carotenoids, compared to the CK.

All treatments enhanced plant growth parameters, with the most significant improvements observed in combined treatments. Si-NPs increased nodules DW by 109.1%, PGPR by 127.7%, and biochar by 130.9%. Trio-combination of these treatments further increased nodules DW up to 159.6%. Similarly, Si-NPs, PGPR, and biochar improved root length by 104.6%, 130.7%, and 147.1% respectively, with the combined treatment reaching 197.8%. For chlorophyll a, Si-NPs led to a 119.2% increase, PGPR to 124.0%, and biochar to 130.1%, with combined treatments achieving up to 209.3%. Chlorophyll b and carotenoids showed the highest improvements with combined treatments, reaching 624.1% and 297.4% respectively. These data specify that the trio-combination of biochar, PGPR, and Si-NPs is highly effective in enhancing plant growth and biochemical attributes.

### 2.5. Changes in Plant-Related Parameters Such as Na and K Contents in Soybean Leaves in Response to Biochar, PGPR and SiNPs

**Figure 6:**
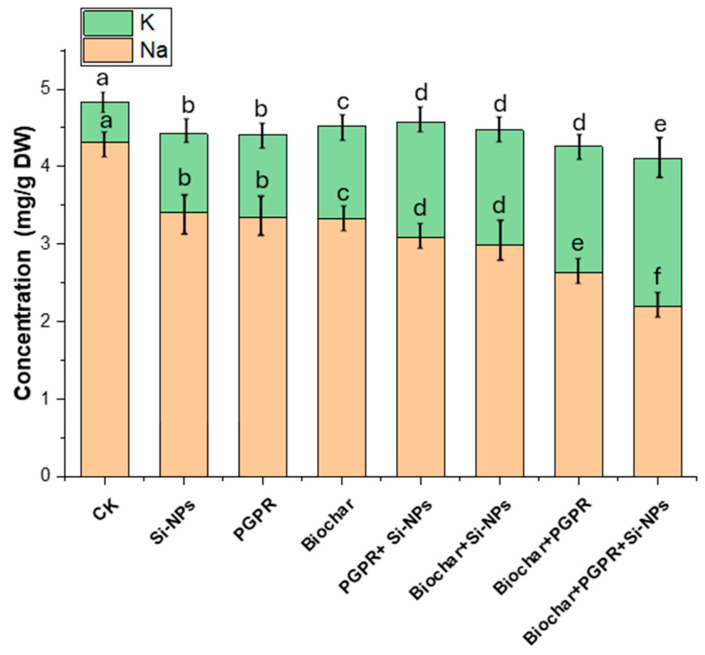
Na and K contents in leaves of soybean (*Glycine max* L.), irrigated with wastewater Cd-polluted and treated with single, coupled and trio-combination of biochar at rate of 10 t ha^−1^, PGPR at rate of (1:1) and Si-NPs (25 mg L^−1^) compared to untreated plants (control; CK). Different letters on bars are significant based on the Tukey’s HSD test (*p* < 0.05).

The results in Figure 6 display the impacts of single, coupled and trio-combination of application biochar, PGPR and SiNPs on soybean plants grown in saline-alkali soil irrigated with Cd-contaminated wastewater. In Figure 6, focusing on the Na and K concentrations in soybean leaves, the CK treatment resulted in a Na concentration of 4.31 mg/g DW and a K concentration of 0.52 mg/g DW. The Si-NPs treatment reduced Na concentration by 20.42% and increased K concentration by 90.38%. PGPR treatment reduced Na concentration by 22.27% and increased K concentration by 101.92%. Biochar treatment declined Na concentration to be 23.04% and a 121.15% increase in K concentration. The coupled of PGPR and Si-NPs reduced Na concentration by 29.93% and increased K concentration by 186.54%. Coupled biochar and Si-NPs reduced Na concentration by 35.97% and increased K concentration by 182.69%. The coupled biochar and PGPR led to a 38.74% reduction in Na concentration and a 211.54% increase in K concentration. The most significant changes were seen in the trio-combination of biochar, PGPR, and Si-NPs, which led to a 49.00% reduction in Na concentration and a 267.31% increase in K concentration compared to CK.

### 2.6. Changes in Antioxidant Enzyme Activity in Soybean Leaves in Response to Biochar, PGPR and SiNPs

**Figure 7:**
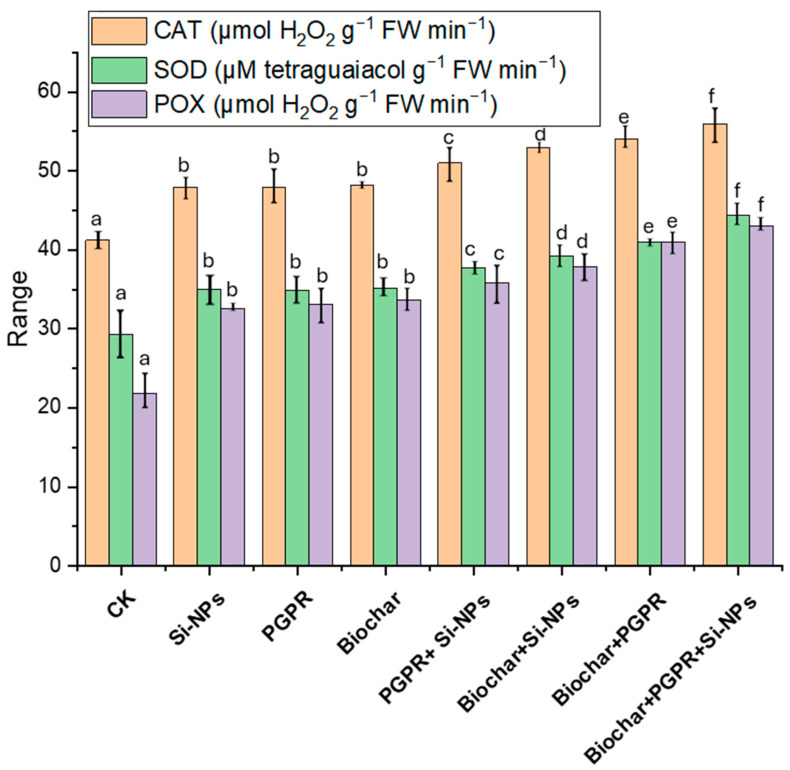
Antioxidant enzymes activity such as catalase (CAT; H_2_O_2_ g^−1^ FW min^−1^), superoxide dismutase (SOD; µM tetraguaiacol g^−1^ FW min^−1^) and peroxidase (POX; µmol H_2_O_2_ g^−1^ FW min^−1^) in leaves of soybean (*Glycine max* L.), irrigated with wastewater Cd-polluted and treated with single, coupled and trio-combination of biochar at rate of 10 t ha^−1^, PGPR at rate of (1:1) and Si-NPs (25 mg L^−1^) compared to untreated plants (control; CK). Different letters on bars are significant based on the Tukey’s HSD test (*p* < 0.05).

The results in Figure 7 reveal the impacts of single, coupled and trio-combination of application biochar, PGPR and SiNPs on soybean plants subjected to saline-alkali soil irrigated with Cd-contaminated wastewater. Figure 7 presents a comprehensive comparison the impact of biochar, PGPR and SiNPs, and their trio-combinations, on the activity of three key enzymes: CAT, SOD, POX. Across all treatments, significant enhancements in enzyme activities were observed compared to the CK, with the most pronounced effects occurring in the combined applications of biochar, PGPR, and Si-NPs. For CAT activity, the CK treatment exhibited an enzyme activity of 39.58 µmol H_2_O_2_ g^−1^ FW min^−1^. The application of Si-NPs resulted in a 19.65% increase to 47.36 µmol H_2_O_2_ g^−1^ FW min^−1^, while PGPR and Biochar treatments yielded increases of 20.16% (47.55 µmol H_2_O_2_ g^−1^ FW min^−1^) and 20.91% (47.85 µmol H_2_O_2_ g^−1^ FW min^−1^), respectively. The trio-combination of PGPR and Si-NPs led to a 27.23% enhancement, resulting in 50.36 µmol H_2_O_2_ g^−1^ FW min^−1^, while biochar combined with Si-NPs and biochar combined with PGPR produced increases of 33.12% (52.68 µmol H_2_O_2_ g^−1^ FW min^−1^) and 35.34% (53.58 µmol H_2_O_2_ g^−1^ FW min^−1^), respectively. The most significant increase in CAT activity, 41.16%, was achieved with the combined treatment of biochar, PGPR, and Si-NPs, reaching 55.85 µmol H_2_O_2_ g^−1^ FW min^−1^.

In terms of SOD activity, the CK treatment showed 26.58 µM tetraguaiacol g^−1^ FW min^−1^. Si-NPs application increased SOD activity by 29.31% (34.36 µM tetraguaiacol g^−1^ FW min^−1^), while trio-combination of PGPR and biochar treatments resulted in 29.99% (34.55 µM tetraguaiacol g^−1^ FW min^−1^) and 31.13% (34.85 µM tetraguaiacol g^−1^ FW min^−1^) increases, respectively. The coupled PGPR and Si-NPs led to a 40.56% increase (37.36 µM tetraguaiacol g^−1^ FW min^−1^), coupled Biochar and Si-NPs as well coupled biochar and PGPR showing enhancements of 45.54% (38.68 µM tetraguaiacol g^−1^ FW min^−1^) and 52.73% (40.58 µM tetraguaiacol g^−1^ FW min^−1^), respectively. The highest SOD activity was recorded in the trio-combination of biochar, PGPR, and Si-NPs a 66.88% increase to 44.35 µM tetraguaiacol g^−1^ FW min^−1^.

For POX activity, the CK treatment exhibited an activity of 19.85 µmol H_2_O_2_ g^−1^ FW min^−1^. Si-NPs application significantly increased POX activity by 61.59% to 32.08 µmol H_2_O_2_ g^−1^ FW min^−1^, while PGPR and biochar treatments resulted in increases of 67.04% (33.15 µmol H_2_O_2_ g^−1^ FW min^−1^) and 68.04% (33.35 µmol H_2_O_2_ g^−1^ FW min^−1^), respectively. The coupled PGPR and Si-NPs produced a 79.65% increase (35.65 µmol H_2_O_2_ g^−1^ FW min^−1^), with biochar combined with Si-NPs and biochar combined with PGPR resulting in 89.70% (37.65 µmol H_2_O_2_ g^−1^ FW min^−1^) and 104.82% (40.65 µmol H_2_O_2_ g^−1^ FW min^−1^) increases, respectively. The highest POX activity, 116.15%, was noted with the combined treatment of biochar, PGPR, and Si-NPs, reaching 42.89 µmol H_2_O_2_ g^−1^ FW min^−1^. These results demonstrate the superior efficacy of combined treatments, particularly the trio-combination of biochar, PGPR, and Si-NPs, in enhancing the activities of antioxidant enzymes. This suggests that these treatments, when applied together, may offer a trio-combination’s effects that strengthen the plant’s defense mechanisms against oxidative stress. The substantial increases in CAT, SOD, and POX activities with the combined treatment underscore the potential of these strategies for improving plant health and resilience, particularly in challenging environmental conditions.

### 2.7. Changes in Oxidative Stress Indicators in Soybean Leaves in Response to Biochar, PGPR and SiNPs

**Figure 8:**
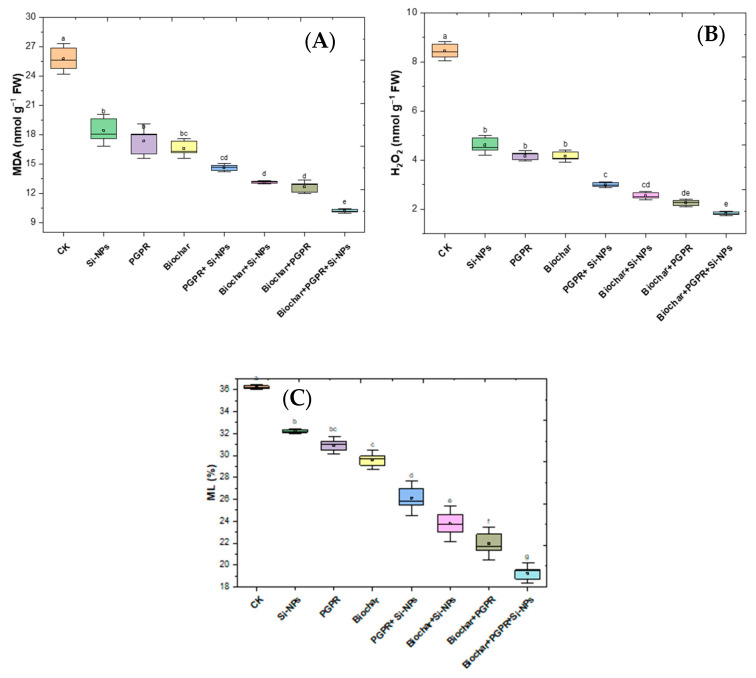
Oxidative stress indicators such as (**A**) lipid peroxidation (MDA; nmol g^−1^ FW), (**B**) hydrogen peroxide (H_2_O_2_; µmol g^−1^ FW) and (**C**) membrane leakage (ML;%) in leaves of soybean (*Glycine max* L.), irrigated with wastewater Cd-polluted and treated with single, coupled and trio-combination of biochar at rate of 10 t ha^−1^, PGPR at rate of (1:1) and Si-NPs (25 mg L^−1^) compared to untreated plants (control; CK). Different letters on bars are significant based on the Tukey’s HSD test (*p* < 0.05).

The results in Figure 8 determine the impacts of single, coupled and trio-combination of application biochar, PGPR and SiNPs on soybean plants grown in saline-alkali soil irrigated with Cd-contaminated wastewater. Figure 8 addresses oxidative stress parameters, MDA, H_2_O_2_ and ML in soybean leaves. CK had MDA levels of 24.78 nmol g^−1^ FW and H_2_O_2_ levels of 8.195 nmol g^−1^ FW. Si-NPs treatment declined MDA at rate of 20.71% and a 40.06% reduction in H_2_O_2_ levels. PGPR treatment further reduced MDA levels by 27.17% and H_2_O_2_ levels by 48.00%. Biochar treatment led to a 29.99% lessening in MDA and a 47.08% reduction in H_2_O_2_ levels. The trio-combination of PGPR and Si-NPs resulted in a 42.20% lessening in MDA levels and a 62.42% lessening in H_2_O_2_ levels. The trio-combination of biochar and Si-NPs reduced MDA levels by 46.85% and H_2_O_2_ levels by 70.20%. Coupled biochar and PGPR together resulted in a 48.03% lessening in MDA levels and a 72.30% lessening in H_2_O_2_ levels. The most significant changes were observed in the trio-combination of Biochar, PGPR, and Si-NPs, which resulted in a 59.32% reduction in MDA levels and a 78.40% reduction in H_2_O_2_ levels compared to CK.

In Figure 8, the osmoprotectant parameter, namely ML (%) was assessed. CK had an ML of 36.35%. The Si-NPs treatment reduced ML by 11.47%. PGPR treatment further reduced ML by 13.94%. Biochar treatment led to a 17.59% reduction in ML. The coupled PGPR and Si-NPs resulted in a 28.97% reduction in ML. Coupled biochar and Si-NPs together reduced ML by 36.69%. The coupled biochar and PGPR showed a 37.26% reduction in ML. The trio-combination of biochar, PGPR, and Si-NPs led to a 48.52% reduction in ML compared to CK treatment.

### 2.8. Changes in Soybean Yield and Related Traits in Response to Biochar, PGPR and SiNPs

**Figure 9:**
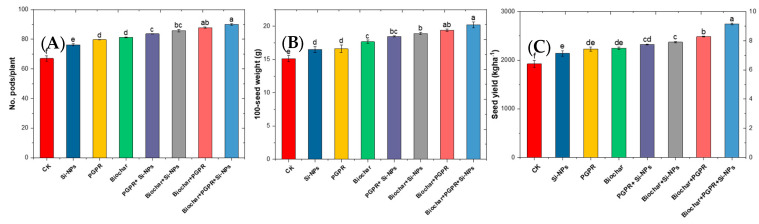
Yield and related traits such as (**A**) number of pods plant^−1^, (**B**) 100-seed weight (g) and (**C**) seed yield (kg ha^−1^) of soybean (*Glycine max* L.), irrigated with wastewater Cd-polluted and treated with single, coupled and trio-combination of biochar at rate of 10 t ha^−1^, PGPR at rate of (1:1) and Si-NPs (25 mg L^−1^) compared to untreated plants (control; CK). Different letters on bars are significant based on the Tukey’s HSD test (*p* < 0.05).

**Figure 10:**
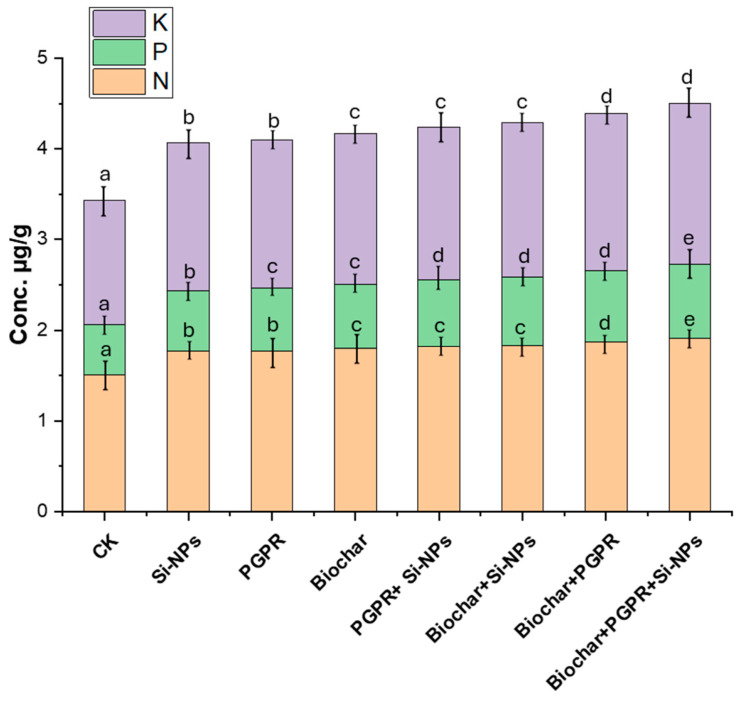
Nutritional value such as N, P and K of soybean seeds (*Glycine max* L.) irrigated with wastewater Cd-polluted and treated with single, coupled and trio-combination of biochar, PGPR at rate of (1:1) and Si-NPs (25 mg L^−1^) compared to untreated plants (control; CK). Different letters on bars are significant based on the Tukey’s HSD test (*p* < 0.05).

The results in Figure 9 determine the impacts of single, coupled and trio-combination of application biochar, PGPR and SiNPs on soybean plants grown in saline-alkali soil irrigated with Cd-contaminated wastewater. Figure 10 illustrates the impact of various treatments—silicon nanoparticles (Si-NPs), PGPR, biochar, and their trio-combination—on key agronomic parameters Compared to the CK, all treatments significantly enhanced these parameters. The CK had 68.86 pods per plant, with Si-NPs increasing this by 9.86% to 75.65 pods. PGPR treatment further improved the number of pods by 15.52% to 79.54, while biochar treatment achieved an 18.62% increase, resulting in 81.69 pods per plant. Combining PGPR and Si-NPs led to a 21.53% increase, yielding 83.69 pods. The coupled biochar and Si-NPs produced a 23.91% rise, reaching 85.36 pods per plant. The coupled biochar and PGPR resulted in a 28.25% increase to 88.36 pods, while the trio-trio-combination of biochar, PGPR, and Si-NPs achieved the most significant improvement of 30.96%, resulting in 90.18 pods per plant.

In terms of 100-seed weight, the CK recorded 15.45 g. Si-NPs application increased the weight by 5.89% to 16.36 g, while PGPR treatment led to a 9.06% increase, achieving 16.85 g. Biochar alone resulted in a 13.77% improvement, reaching 17.58 g. The coupled of PGPR and Si-NPs increased the 100-seed weight by 20.06% to 18.55 g, while biochar and Si-NPs together yielded a 22.88% rise, bringing the weight to 18.98 g. Coupled biochar with PGPR showed a 24.39% increase, resulting in 19.22 g. The highest 100-seed weight, a 31.78% improvement, was recorded with the trio-combination of biochar, PGPR, and Si-NPs, resulted in 20.36 g. Regarding seed yield per hectare, the CK produced 1849.19 kg ha^−1^. Si-NPs increased the yield by 17.04% to 2164.83 kg ha^−1^, and PGPR treatment further improved it by 19.79% to 2215.83 kg ha^−1^. Biochar treatment achieved a 21.04% increase, reaching 2237.83 kg ha^−1^. Coupled PGPR and Si-NPs resulted in a 26.28% rise, yielding 2334.83 kg ha^−1^. The coupled biochar and Si-NPs produced a 28.58% increase, achieving 2377.83 kg ha^−1^, while biochar and PGPR together resulted in a 34.78% rise, reaching 2491.83 kg ha^−1^. The highest yield, a 47.34% increase, was observed with the trio-combination of biochar, PGPR, and Si-NPs, resulting in 2725.19 kg ha^−1^. The consistent superior performance of treatments, especially the trio-combination of biochar, PGPR, and Si-NPs, underscores their synergistic effect and highlights their potential to significantly and sustainably boost agricultural productivity.

### 2.9. Changes in Nutritional Value of Soybean Seeds in Response to Biochar, PGPR and SiNPs

The results presented in Figure 10 assess the effects of single, combined, and trio applications of biochar, PGPR, and Si-NPs on soybean plants cultivated in saline-alkali soils irrigated with Cd-contaminated wastewater. The effects of silicon nanoparticles (Si-NPs), PGPR, biochar, and their coupled and trio-combination on soybean seed nutrient content (N, P, K) were significant when compared to CK values. Si-NPs alone increased N by 12.5%, P by 34.7%, and K by 13.8%. PGPR treatment led to a greater rise in nutrient levels, with N up by 16.4%, P by 42.9%, and K by 18.1%. Biochar alone showed a 17.8% increase in N, 46.9% in P, and 19.6% in K. The coupled PGPR and Si-NPs further elevated N, P, and K by 19.7%, 51.0%, and 21.7%, respectively. Coupled biochar and Si-NPs resulted in increases of 21.1% in N, 53.1% in P, and 23.2% in K. Coupled biochar and PGPR showed the highest single-treatment improvement, with N up by 22.4%, P by 57.1%, and K by 24.6%. The most noted with the trio-combined treatment of biochar, PGPR, and Si-NPs, where N increased by 25.0%, P by 65.3%, and K by 27.5%. This data highlights the substantial potential of these treatments to enhance soybean seed nutrient content, especially when trio-combined. Each treatment individually improved nutrient levels compared to the CK, with biochar often yielding slightly better results than Si-NPs or PGPR alone. The trio-combination consistently outperformed single treatments, underscoring the trio-combination of effects of using biochar, PGPR, and Si-NPs together, which could lead to improved crop yield and nutritional value.

### 2.10. Analysis of Principal Component Analysis (PCA)

**Figure 11:**
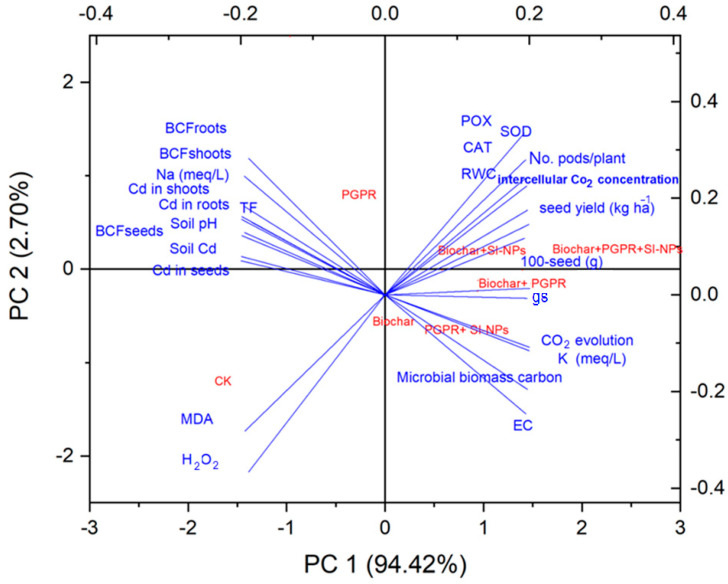
PCA of individual response variables determined in soybean (*Glycine max* L.) irrigated with wastewater Cd-polluted and treated with single, coupled and trio-combination of biochar, PGPR at rate of (1:1) and Si-NPs (25 mg L^−1^) compared to untreated plants (control; CK).

The PCA presented in Figure 11 provides a comprehensive analysis of the effects of the various treatments. PC1 accounting for 94.42% of the total variance, reveals distinct clustering of treatments based on their influence on plant growth, nutrient uptake, and stress tolerance. Treatments involving coupled Si-NPs and PGPR, consistently exhibit positive loadings on PC1. These treatments appear to enhance nutrient uptake, as indicated by higher levels of sodium (Na) and potassium (K) in plant tissues. Furthermore, these treatments may improve plant stress tolerance, as evidenced by lower levels of malondialdehyde (MDA), a marker of oxidative stress. Biochar, while not as potent as Si-NPs and PGPR, also shows positive loadings on PC1, suggesting some beneficial effects on plant growth and physiology. However, biochar alone seems less effective than when combined with other treatments.

PC2 accounting for 2.70% of the total variance, primarily differentiates treatments based on their impact on plant root physiology and microbial interactions. Treatments involving biochar and PGPR tend to have higher loadings on PC2, indicating their influence on root growth, microbial biomass carbon, and CO_2_ evolution. Overall, the PCA analysis suggests that the coupled Si-NPs and PGPR is particularly effective in promoting plant growth and stress tolerance. These treatments appear to enhance nutrient uptake, reduce oxidative stress, and stimulate microbial activity in the rhizosphere. While biochar also had some positive effects, its impact is less pronounced compared to Si-NPs and PGPR. The PCA analysis provides valuable insights into the complex interactions between different treatments and their impacts on plant growth and physiological parameters. The findings suggest that the trio-combination of biochar, PGPR and Si-NPs can be a promising strategy for improving plant productivity and resilience in challenging environmental conditions.

## 3. Discussion

### 3.1. Effect of Co-Applied Biochar, PGPR and SiNPs on Soil Chemical and Biological Indicators Under Cd-Polluted Saline Soil Irrigated with Wastewater

Cadmium-contaminated, salt-affected and wastewater-irrigated soils are coomon in agricultural lands depending on the Kitchener Drain for irrigation of crops in northern Egypt. These conditions pose a significant threat to soil quality, severely affecting soil chemical properties, root growth and nutrient uptake, which in turn negatively impacts plant growth and productivity [16]. Hence, there is a need for innovative approaches to mitigate the inevitable accumulation of Cd and Na in soil, allowing plants to grow in a healthy and sustainable environment while reducing the transfer of hazardous elements to different tissues. This investigation introduce a comprehensive understanding of how soil amendments such as biochar and PGPR, combined with foliar application of Si-NPs can be exploited to enhance soil chemical properties, microbial activity, enzyme activity and antioxidant enzyme function. These interventions are expected to improve plant growth, physiological processes, increase the amount of assimilates transported from nutrient organs to seeds, and consequently increase yield-related traits and seed nutritional value in soybean plants growing in heavy metal contaminated and salt-affected soils., providing sustainable agricultural practices and tackle the growing food demands. Previous studies have shown that Cd-contaminated and salt-affected soils typically impair root development, reduce soil biological activity, and hinder basic cellular metabolism [16] However, pther researches [32,42] have highlighted the positive impact of soil applications such as biochar and PGPR as well as foliar sprays such as Si-NPs, in improving soil properties and physiological processes, thereby helping plants cope withenvironmental stresses. While these studies have provided valuable insights, they have not focused on the combined effects of cadmium contamination, salinity, and wastewater irrigation. Moreover, the potential of using a trio-combination of biochar, PGPR, and Si-NPs to mitigate Cd and Na stress in soybean plants has not been well explored. Given thatsoybean is an imperative crop among cultivated grain legumes, augmenting its production is of worldwide importance. This study, therefore, evaluates the effect of biochar, PGPR and Si-NPs individually and in combined on soybean plants grown in Cd-polluted, saline soils irrigated with wastewater.

In this study, Cd-contaminated soil, salt-affected soil and increased reliance on wastewater for irrigation collectively triggered significant disturbances in soil chemical properties, such as elevated pH, EC, Na content and ESP (Figure 1) These changes negatively affected soil microbial activity, as evidenced by decreased soil CO_2_ evolution and reduced microbial biomass carbon content (Figure 3), in line with results from [14]. In addition to diminished microbial activity, soil enzyme activities, such as urease, dehydrogenase, and alkaline phosphatase, were also reduced (Figure 2), consistent with previous research by [11]. Soil degradation resulting from Cd-polluted saline soil and irrigated with wastewater led to a decline in root parameters, such as root length and nodules weight (Figure 5) as well an increase in Cd and Na ion concentrations in plant parts. This, in turn, caused heightened oxidative stress as indicated by increased levels of (H_2_O_2_ and MDA),and distubed physiological and biochemical processes. eventually impairing yield-related traits in soybean plants (Figure 10). These results are in agreement with [12]. Given these results, the importance of our study becomes evident. It is clear that the applying of biochar with PGPR and SiNPs, represents a promising trio-combination to improve plant resilience and survival in Cd-polluted saline soils irrigated with wastewater.

When biochar was applied to soil, it significantly improved soil chemical properties such as neutralizing soil alkalinity by lowering pH, enhancing nutrient availability and boosting microbial activity compared to untreated plots [14,26,27]. Studies by [43,44] demonstrated that biochar stabilizes soil aggregates, increases soil air-water balance, and enhances hydraulic conductivity, as well as nutrient/water-holding potential of the soil. The unique chemical composition of biochar, which includes inorganic elements such as Na, K, Mg, Ca, Si, increases the nutritional acquisition of plants from the soils [45]. Additionally, biochar includes surface functional groups which offer crucial sites for the adsorption of metals, including Cd and Na, as well as alterations to cation exchange sites [25]. Biochar positively influences soil permeability and membrane stability index, as noted by [10]. It ahs also been demonstrated that biochar can decline the ion toxicity such as cadmium and ion imbalance (i.e., higher K/lower Na). As a result, biochar amendment reduced Cd bioavailability and uptake, effectively immobilizing Cd toxic in the soil [15].

Many studies have highlighted the positive impacts of biochar, particularly its ability to improve water retention around the rhizosphere [46]. Biochar residues can stabilize soil pollutants, such as Cd, and help maintain ionic balance by lowering soil pH, which in turn increases K ions availability, providing essential nutrients for plant growth [47]. These multifaceted benefits can be further improved when biochar co-applied with PGPR along with foliar spray of SiNPs. Incorporating biochar and PGPR has significantly greater impact on Cd and Na levels in the soil compared to their individual use, positively influencing soil health, moisture retention, and root growth. According to [48], increasing the soil moisture holding capacity around the rhizosphere gives soybeans a greater withstand soil salinity, reducing Na flux and enhancing root activity and nutrient uptake. This leads to improved gas exchange and overall plant performance. Additionally, it has been observed that the combined use of biochar with PGPR enhance PGPR efficacy, as biochar provides a suitable substrate with its high surface area and essential nutrients that support the long-term survival of PGPR. Study of [46] demonstrated that co-applied of biochar with PGPR improved water holding capacity by 24% and 18% compared to PGPR solely and biochar solely. Furthermore, ref. [49] pointed out that co-applied biochar with PGPR augmented the availability of N, P, and K by 73%, 173%, and 17%, respectively compared to the individual application. Co-applied of biochar and PGPR significantly increased soil enzymes activity, which in turn enhanced soil biochemical processes and nutrient equilibrium, thereby improving soil health. Also, it was noticed that PGPR solubilized organic nutrients and accelerated the release of nutrients from biochar.

Thus, the co-applied of PGPR and biochar not only facilitates availability of nutrients but also triggers improved enzymatic activity [25,50]. Overall, it was confirmed that biochar co-applied with *Bradyrhizobium japonicum* (USDA 110) and *Trichoderma harzianum* can improve various soil physicochemical properties, enhance soil biological indicators, and ultimately promote nodulation, root growth and symbiotic interactions, as reported by [51]. The authors [45] attributed these results to enhanced soil carbon dioxide evolution and increased soil microbial biomass content, which provided more oxygen for nodule bacteria, boosting their ability to colonize roots and maintain a long term symbiotic relationship. Additionally, PGPR co-applied biochar produce plant hormones, such as IAA and gibberellins, that promote root length and nodule formation, ultimately improving soil microbial community structure, nutrient content and ion flux balance [20].

### 3.2. Effect of Co-Applied Biochar, PGPR and SiNPs on Translocation of Cadmium and Sodium Ions from Soil to Soybean Plant Parts Under Cd-Polluted Saline Soil Irrigated with Wastewater

Cd-polluted saline soil irrigated with wastewater has been shown to increase soil Cd and Na content, which negatively affects soil health by reducing soil respiration, altering chemical properties and decreasing soil enzymes activity. Furthermore, this condition disturbs soil microbial activity. The increased presence of sodium ions in the soil competes with potassium ions, leading to a reduction in plant development. Additionally, the solubility of Cd ions is enhanced, leading to greater absorption by the plant roots and subsequent transfer to various plant parts, which interferes with essential plant processes [12]. Some studies have indicated that the application of PGPR with biochar can reduce Na uptake and increase K/Na ratio in the soil, thereby limiting Na uptake in the xylem and increasing K^+^ availability [44]. Our results showed a significant reduction in ESP and Na in soil solution, alongside increased K^+^ uptake when biochar was applied with PGPR (Figure 6). This led to an improvement in the K^+^/Na^+^ ratio (Figure 6). The co-application of PGPR with biochar, which promotes exopolysaccharides production and increases microbial colonization efficiency, had a positive impact by binding Na^+^ in the soil [46,52]. These findings align with previous research showing that PGPR co-applied with biochar helps sustain nutrient equilibrium by releasing an essential elements like K, Ca and Mg. This process effectively reduces Na ions in the soil, thereby increasing the K^+^/Na^+^ ratio and balancing ion influx [17,47].

The observed increasein the bioremediation of Cd from the soil through the coupled PGPR with biochar can be partially explained by the increased soil enzymes activity (urease, dehydrogenase and alkaline-phosphatase). These enzymes are released by nitrogen fixing bacteria and phosphate-solubilizing bacteria, which contribute essential nutrients to the soil solution (Figure 2) as noted by [53]. Furthermore, PGPR co-applied with biochar are known to reduce soil pH and make K more available to plants, which in turn helps reduce Cd-toxicity by roots (Figure 1) as mentioned by [42]. The coupled treatment also declined HOAc-extractable Cd levels compared to the individual application of either biochar or PGPR, as mentioned by [43]. evidence suggests that the presence of PGPR in the presence of biochar regulate soil pH, providing inoculants with a more favorable habitat its richness in carbon, porous structure of biochar. This enhances microbial longevity and promotes the growth of beneficial bacteria. Biochar’s high surface area also facilitates the release of ACC deaminase, the secretion of metabolites, the production of exopolysaccharides, and the synthesis of phytohormones, all of which reduce ethylene production and enhance microbial colonization in the plant rhizosphere [54]. These processes contribute to the immobilization of Cd through adsorption and remediation, effectively reducing its availability in the soil [16].

Although SiNPs was applied to the aboveground parts of the plants, they also have the potential to enhance the plant rhizosphere in combination with PGPR and biochar under Cd-polluted saline soil irrigated with wastewater, as compared to CK treatment (Figure 3). Sprayed SiNPs can enter plant roots through stomata via phloem which follows the hydrophilic pathway, facilitated by membrane transporters and carrier proteins [33,55]. It has been noted that foliar spraying with SiNPs can alleviate Cd and Na contents in the leaves, as well as in the soil, when dealing with Cd-polluted saline soil irrigated with wastewater. This treatment helps maintain K^+^/Na^+^ homeostasis and improves BAC, TF and BAC (Figure 4) as noted by [56].

These results demonstrate that SiNPs, when used in a trio-combination with PGPR and biochar, induced tolerance to Cd-polluted saline soil irrigated with wastewater. Our findings align with those of previous studies [33,57], which highlighted the spherical shape and nanosize of SiNPs as key features that enable them to easily penetrate through stomata and be effectively transported via membrane carriers to different parts of the plant [58]. Remarkably, our findings confirm that foliar SiNPs application lessened Cd concentration of each part while maintaining K^+^/Na^+^ homeostasis thereby facilitating the transport of nutrients for plant growth [59]. Moreover, our study shows that co-applied of PGPR with biochar in presence of foliar SiNPs, significantly improved soil quality and remediated Cd-polluted saline soil irrigated with wastewater, outperforming sole application. Study by [52] observed that PGPR co-applied with biochar alleviated salt stress and enhanced plant growth by enhancing K^+^/Na^+^ ratio. However, study [44] found that PGPR co-applied with biochar reduced Cd availability by 45.6% compared to individual treatment. These processes work synergistically to mitigate metal toxicity, improving soil health and supporting plant growth [47].

### 3.3. Effect of Co-Applied Biochar, PGPR and SiNPs on Physiological Processes, Antioxidant Enzymes, Oxidative Stress and Osmoprotectants in Soybean Leaves Under Cd-Polluted Saline Soil Irrigated with Wastewater

In terms of physiological processes, it was observed that soybean plants exposed to saline soil contaminated with Cd and irrigated with wastewater exhibited degradation of chlorophyll and disruption of gas exchange processes. This was attributed to the inhibition of antioxidant enzymes activity and increased oxidative stress, which in turn affected ion exchange, damaging chloroplasts, reducing chlorophyll synthesis, and causing membrane leakage. These changes led to the disruption of physiological and biochemical processes (Figure 7 and Figure 9). Elevated levels of Ca and Na ions in plant tissues boosted ROS production, particularly H_2_O_2_ and O_2_^−^. Concurrently, CAT, SOD and POX decreased (Figure 7), resulting in higher levels of MDA, H_2_O_2_ and EL (Figure 8).

Our results indicate that the biochar co-applied with PGPR and SiNPs significantly mitigated the negative effects of Cd-contaminated saline soil irrigated with wastewater. This trio-combination reduced oxidative stress, increased antioxidant enzymes activity, and improved photosynthesis, thereby supporting better physiological and biochemical processes and promoting dry matter transport in soybean plants (Figure 7 and Figure 8). Additionally, the trio-combination application improved leaf water status, gas exchange processes and chlorophyll pigments, likely by increasing K and Mg levels in leaf tissues [60]. These essential nutrients also play a crucial role inphotosynthesis, with K and Mg acting as cofactors for key enzymes that increase the thickness of chloroplasts and thylakoid membranes. Additionally, they help reduce the accumulation of organic metabolites, such as proline and glycine betaine, which improve the plants ability to survive under Cd-contaminated saline soil irrigated with wastewater. This eventually supports better metabolism and physiological processes [61].

The co-applied of PGPR with biochar significantly enhanced soil chemical and biological properties, having a more pronounced impact than SiNPs in reducing bioavailability and uptake of Cd and Na, thereby enhancing nutrient availability in the rhizosphere. However, as an elicitor, SiNPs strengthened the antioxidant defense system, which reduced the bioavailability and uptake of Cd and Na in the leaves [57,62,63]. This, in turn, enhanced photosynthetic CO_2_ assimilation and maximized nutrient uptake, resulting in a more significant than co-applied PGPR with biochar [59,64]. This promotes our presupposition that trio-combination application of biochar with PGPR and SiNPs to leaves and soil is much better compared than individual treatments, as it helps bypass potential saturation conditions which could take place either in the roots or the leaves. Thus, it was confirmed that PGPR co-applied biochar, in the presence of SiNPs, enhances crop tolerance to saline soil contaminated with Cd and irrigated with wastewater. Previous studies have shown that K ions are absorbed in place of Na ions, resulting in increased nutrient uptake and higher relative water content [52]. Another study [65] found that microbial inoculants (Bacteria and fungi) promoted the immobilization and reduced uptake of HMs, which in turn increased nutrient uptake. The study by [58] suggested that foliar spraying with SiNPs upregulated antioxidant enzymes activity, stimulated cell elongation and preserved membrane integrity, thereby improving structure of chloroplast, increasing physiological and biochemical processes under Cd stress. A recent study by [66] implied that foliar spraying with SiNPs declined Cd uptake and transport in rice by improving polysaccharide metabolism and cell wall maintenance. Furthermore, it was shown that foliar spraying with SiNPs scavenged ROS and alleviated oxidative damage (MDA and H_2_O_2_) in wheat and canola by augmenting SOD, CAT and POX which maintained nutrient balance under cadmium stress [55,56].

### 3.4. Effect of Co-Applied Biochar, PGPR and SiNPs on Yield-Related Traits and Nutrient Content in Soybean Seeds Under Cd-Polluted Saline Soil Irrigated with Wastewater

Regarding yield-related traits and nutrient content in soybean seeds, it was observed that soybean plants exposed to saline soil contaminated with cadmium and irrigated with wastewater exhibited significant degradation in yield-related traits (pods number plant^−1^, 100-seed weight and seed yield) (Figure 9), as well as a reduction in nutrient content (N, P and K) (Figure 10). The presence of cadmium in the saline soil, coupled with wastewater irrigation, led to reduced water uptake, which in turn decreased nutrient absorption and disrupted photosynthetic assimilation [67]. Specifically, Cd-contaminated saline soil irrigated with wastewater significantly impaired the potential of soybean plants to uptake water, which led to a decrease in the absorption of the necessary nutrients needed to transport the assimilation of photosynthesis from the source to the sink [38]. This disturbance in nutrient transport ultimately led to a decline in photosynthetic activity and nutrient transfer [40,49], contributing to reduced pod fullness, pod number, and final crop yield, along with a decrease in the concentration of essential nutrients in the soybean seeds [3,17]. Maintaining both high quality and quantity of soybean crop requires increased nutrient flow, as well as improved fitness, performance, and survival under conditions of saline soil contaminated with cadmium and irrigated with wastewater, a highly desirable trait [14]. In this study, untreated plots (CK) exhibited the lowest yield related traits (Figure 9) and nutrient content in soybean seeds (N, P and K) (Figure 10). However, the trio-combination application of biochar with PGPR in the presence of SiNPs resulted in significantly higher productivity and seed quality than a single application or binary combination. The trio-combination effect achieved the highest augment in yield and seed quality due to the combined application of soil amendment, which improved the availability and uptake of nutrients from soil to plant parts, and also promoting root growth and improving metabolic functions [3,16,23]. Additionally, the combined of soil application enhanced microbial biomass and soil enzyme activity, thus improving soil quality [47]. Foliar application of SiNPs, on the other hand, contributed to ion exchange balance (K^+^/Na^+^), strengthened theantioxidant defense, and reduced oxidative stress (Figure 8). This supported photosynthesis and the remobilization of pre-anthesis accumulated substances, further improving physiological and biochemical processes [44]. The trio-combination provides a stronger defense against oxidative stress induced by Cd and Na, improving soil health (through Cd immobilization and Na binding) [67] and increasing nutrient cycling (N, P, and K) [37]. This balance regulates redox status, and preserves cell membrane lipid integrity [61,67], supporting photosynthesis and remobilization of accumulated substances to promote better plant growth, development, seed productivity, and nutritional value in soybean plants exposed to saline soil contaminated with Cd and irrigated with wastewater. The combined use of silicon nanoparticles (Si-NPs), biochar, and plant growth-promoting rhizomicrobes (PGPR) holds great promise for enhancing soil health, reducing heavy metal bioavailability, and boosting plant growth, with immense potential for sustainable agriculture. Biochar’s stability ensures long-lasting benefits by improving soil structure, nutrient retention, and microbial activity, while Si-NPs contribute to strengthening plants against stress through enhanced silicic acid availability. PGPR actively supports nutrient cycling, enzyme activity, and soil microbial health, fostering a resilient ecosystem. Together, these amendments work synergistically to improve soil quality and productivity, while mitigating environmental stresses. To maximize these benefits and ensure sustainable application, long-term studies and optimization of application practices are recommended, enabling us to harness their full potential while safeguarding soil health and ecosystem balance.

## 4. Materials and Methods

### 4.1. Depiction of the Trial Location

The soybean plants were grown in the experimental field of Kafr El-Sheikh governorate, located in north Egypt (31.40° N, 31.17° E) during summer 2023. This zone is categorized with Subtropical weather and the irrigation water of kitchener drain was the major supply utilized which characterized in (Table 1).

The permissible level of Cd, Pb and Ni in irrigation water recommended by FAO is 0.01, 0.1 and 0.2 mg L^−1^ [2]; So, the utilized irrigation water in the current investigation is described as Cd-contaminated irrigation whereas Cd content is roughly 9-fold > the normal. The soil characters in the present investigation are described as clayey soil with the next physical and chemical characteristics as analyzed by lab of soil science, Agricultural Research Center, Giza as characterized in (Table 2).

Cd content relies on soil structure; so, the highly recommended soil Cd content differs due to soil properties. Therefore, soils with higher clay content show greater Cd content [3]. Whereas the soil is clayey with a Cd content of 5.15 ± 0.22 mg kg^−1^, it is described as Cd-contaminated soil. Also, the continuous consumption of Cd-contaminated irrigation water would convert to Cd-contaminated soil. The acceptable limits of Cd, Pb, and Ni in soil based on the European Union Standards are 3, 300, and 75 mg/kg, respectively [4].

### 4.2. Experimental Layout, Treatments, and Growth Conditions

The investigation was distributed using a Completely Randomized Factorial layout with eight treatments as follows: control (CK), biochar, PGPR, silicon nanoparticles (SiNPs), coupled of biochar + SiNPs, coupled of PGPR+ SiNPs, coupled of biochar+ PGPR and trio-combination of biochar + PGPR + SiNPs and every experimental condition were replicated three times. A mixture of two plant growth-promoting rhizomicrobes (PGPR) strains (1) the bacteria (*Bradyrhizobium japonicum* (USDA 110) and (2) the fungi (*Trichoderma harzianum*) which were selected according to their potential to salinity and Cd tolerance then seed inoculated at percent (1:1) and rate of 950 g ha^−1^ before sowing. Biochar was used as a soil amendment broadcasting before the second ploughing at a rate of 10-ton ha^−1^ [16,68]. SiNPs was attained from Division of Faculty of Agriculture, Azhar University, Egypt. The SiNPs (25 mg L^−1^) suspension was foliar applied on the top of soybean plants at a rate of 400 L ha^−1^ at 20, 35, 50 and 65 DAS using a small handheld sprayer [69] which was described to have a purity of 99.5%, particle size of 10–20 nm, surface area of (270–330 m^2^ g^−1^), pH of (4.0–4.5) and average diameter of (10 nm). Whilst plots not sprayed with SiNPs were treated with an equivalent amount of distilled water. Soybean seeds (*Glycine max* L. cv. Giza 111) were purchaced from Division of Leguminous Crops, Agricultural Research Center, Egypt and were cultivated at a rate of 95 kg seed ha^−1^ on 2 June 2023. Seeds were cultivated in hills at a rate of 2–4 seeds per hole with 25 cm distance on one ridge. The experimental unit was 20 m^2^ (4 × 5 m) and it included 8 rows with a spacing of 50 cm between rows. Irrigation was executed with Kitchener drain water. Soybean plants irrigated five times with 21-day intervals starting from sowing till harvesting.

#### Sources and Analysis of Biochar

Biochar used in the current investigation was set by pyrolysis of two residual plants (Table 3), i.e., rice husk and corn stalk, at a ratio of 1:1 at 500 °C for 3 h in a muffle furnace in hypoxia [16].

### 4.3. Measurements

#### 4.3.1. Soil Chemical Properties Measurement

At harvesting, soil samples were selected by an auger from each plot. Then, air dried and tightly cleansed by passing it through a 2-mm sieve and reserved in polyethylene sacks. pH was measured by pH-meter (Genway, Cambridgeshire, UK, relative error; ±0.05) meanwhile The ECe (dS m^−1^) was assessed by EC-meter (Genway, UK). The levels of Na^+^ and K^+^ ions (meq L^−1^) were assessed by (AAS, Perkin Elmer 3300, Shelton, CT, USA) with a limit of detection (LOD) as low as 100 ppb [5]. Soil exchangeable Na^+^ % (ESP) were estimated by measured Na^+^, K^+^, Ca^2+^, and Mg^2+^ ions [70].

#### 4.3.2. Soil Enzymes Activity

The soil’s nitrogenase, dehydrogenase and alkaline-phosphatase activities were measured by selecting soil samples at 80 days from each experimental plot for estimating soil enzyme, which cleansed carefully and passed through a 2-mm sieve then put in an aseptic polyethylene bag and put in an ice storage box (in a −20 °C freezer) to prepare it for analysis in laboratory. soil’s urease activity (µg TPF g^−1^ dry soil 24 h^−1^) was measured by the quantitative determination of ammonia by the spectrophotometric by [71]. The soil’s dehydrogenase activity (µg TPF g^−1^ dry soil 24 h^−1^) was measured by [6]. The soil’s alkaline-phosphatase activity (mg phenol g^−1^ dry soil 24 h^−1^) was measured by p-nitrophenyl phosphate, and the finding color intensity was assessed by colorimetric technique [7].

#### 4.3.3. Soil Microbial Indicators

Microbial biomass content was measured as an indicator of soil physical stability and nutrient cycling. So, it may be deemed an appropriate biological indicator of soil quality. Soil Microbial Biomass Carbon (SMBc; mg g^−1^ Soil) was assessed at 80 days by the fumigation incubation-extraction technique according to [72]. It was selected 25 g soil samples, then stored overnight at 25 °C with ethanol-free chloroform. Then, it was added 0.5 M K_2_SO_4_ (100 mL) on the fumigated and nonfumigated sample flasks and shaking for 30 min. The solutions are filtered through acid-washed Whatman no. 42 filter paper. The filtrates were extracted with 0.5 M K_2_SO_4_ (100 mL) for 30 min. After that, the organic C concentration was measured by K_2_Cr_2_O_7_ and concentrated with ferrous ammonium sulphate in tubes and weighted then put in autoclave for 30 min at 170 °C, based on [73].

Soil CO_2_ evolution measurement (mg CO_2_ 100 g^−1^ soil 24 h^−1^) as soil microbial activity. Soil samples were grounded and sieved to a size of less than 2 mm for typical use according to Coleman et al. [74]. 50 g of soil were placed in an incubator under a soil moisture regime of 70% water holding capacity at 25 °C into Erlenmeyer flasks (1 L capacity). Next, the soil sample is placed in a beaker containing NaOH was then added with a 0.10 M HCl solution to reach pH 8.3 and the evolution of carbon dioxide in the soil was then measured.

#### 4.3.4. Extractable Soil Cd Content (mg kg^−1^)

At harvest time, soil samples (0–20 cm) were selected and sieved to be typically used at 2 mm then kept at −20 °C for measure of extractable soil Cd content which were extracted by EDTA according to the method of Manouchehri et al. [75] and quantified by (AAS, PerkinElmer 3300, Shelton, USA) [8].

#### 4.3.5. Cd Content in Different Plant Tissues

At harvest, five plant samples were selected from each experimental unit. Each sample was detached into root, shoot, and seeds then cleansed and tightly air-dried then put in oven at 70 °C for 24 h. Crashed them by a bead grinder (model: EDW-50, Shanghai, China) to a fine powder and reserved them in polyethylene sacks for any required analyses. Root samples were rinsed with 1M HCl and afterward carefully bathed in deionized water. A 500 mg fine powder of plant tissue was combined with 4.0 mL HNO_3_ and 1.0 mL HClO_4_ then digested in a 25-mL Kjeldahl digestion tube and filtered to obtain a clear solution. Samples were put in oven to 150 °C for an hour. Cadmium concentration was assessed via flame atomic absorption spectroscopy (AAS PerkinElmer 3300, Shelton, USA) [9]. The Cd content efficiency of soybean plants was determined for each plant tissue. Bioconcentration factor (BCF), translocation factor (TF), and bioaccumulation coefficient (BAC) from soil to aboveground tissues were estimated as follows [31,76,77]:

$$BCF=\frac{Cd\;content\;in\;plant\;root}{Cd\;content\;in\;soil}$$

$$TF=\frac{Cd\;content\;in\;plant\;shoot}{Cd\;content\;in\;plant\;root}$$

$$BAC=\frac{Cd\;content\;in\;plant\;shoot}{Cd\;content\;in\;soil}$$

#### 4.3.6. Root Related Traits

At 80 DAS, it was collected five plants randomly from each experimental unit. after cleansed it from any soil then measured it’s length (cm). For nodules also separated c from roots then measured it’s weight (mg plant^−1^).

#### 4.3.7. Chlorophyll Pigments Measurement

Chlorophyll a, b, and carotenoids were assessed at 80 days based on [78]. Five fully developed top leaves were cleansed. So, 2 g of the leaves were attained and the absorbance was assessed at 663, 645, and 470 nm by a spectrophotometer. Contents of photosynthetic pigments were assessed as mg g^−1^ FW as follows:

Chl a (mg g^−1^ FW) = 12.25A662.9 − 2.78A650.9

Chl b (mg g^−1^ FW) = 21.50A650.9 − 5.10A662.9

Carotenoids (mg g^−1^ FW) = 1000A470 − 1.82Chl a − 85.02Chl b

#### 4.3.8. Ionic Homeostasis (Na^+^ and K^+^)

At 80 days, it was collected five fully developed top leaves randomly in each experimental unit afterwards cleansed then ovened at 65 °C for 72 h until stable weight. Combined acids HNO_3_:HClO_4_ (2:1 *v*/*v*) for 120 min at 220 °C were used to digest a 200 mg dried sample after putting them in 25 mL Kjeldahl tube. Na^+^ and K^+^ contents in the digested samples were measured by (AAS PerkinElmer 3300, Shelton, USA) based on technique of [79].

#### 4.3.9. Antioxidant Enzymes Activity

For the measurement of CAT, SOD and POX enzymes activity, it was collected five fully developed top leaves from each experimental unit at 80 DAS. The Catalase activity (CAT: 1.11.1.6; µmol H_2_O_2_ g^−1^ FW min^−1^) was detected using the reaction between 50 µL enzyme extract and 12.5 mm H_2_O_2_ in the attendance of 50 mM K-phosphate buffer (pH 7.0). The reaction initiated using applying H_2_O_2_ and the absorbance was investigated at 240 nm for 60 s [80]. Superoxide dismutase activity (SOD: 1.15.1.1; µM tetraguaiacol g^−1^ FW min^−1^) was evaluated using nitro-blue tetrazolium (NBT) photochemical assay at 560 nm as illustrated by [81]. Peroxidase (POX: 1.11.1.7; µmol H_2_O_2_ g^−1^ FW min^−1^) activity was evaluated via o-phenylenediamine as a chromogenic factor in the existence of H_2_O_2_ and enzyme extract at 417 nm as depicted [82].

#### 4.3.10. Oxidative Stress Indices

For the measurement of MDA and H_2_O_2_, it was collected five fully developed top leaves from each experimental unit at 80 DAS. MDA (nmol g^−1^ FW) was estimated via the thiobarbituric acid test (TBA) by [83]. Briefly, 200 mg of tissues was mixed and milled in liquid nitrogen with hydro-acetone buffer (4:1 *v*/*v*). So, 20% trichloroacetic acid (TCA) solution and 0.01% butyl hydroxyl toluene (BHT), and the samples were then incubated at 95 °C. centrifuged at 10,000× *g* for 10 min. The absorbance was estimated spectrophotometrically at 532 nm and 600 nm via the model UV-160A (Shimadzu, Kyoto, Japan). H_2_O_2_ (µmol g^−1^ FW) in 1 g of samples was estimated using [84]. The solution extraction contained via liquid nitrogen and TCA (0.1%), next by centrifugation at 6000× *g* for 15 min. The content of the yellow color in the supernatant was then computed at 426 nm by the model UV-160A spectrophotometer (Shimadzu, Kyoto, Japan).

Membrane leakage was measured by putting 1 cm^2^ leaf discs in sample tubes containing 10 mL of deionized water. ML was computed by the equation: $ML\;(\%)=\frac{C1}{C2}\times 100$ [10], C1 expresses EC of samples put in a water soak at 55 °C for 25 min, and C2 expresses the EC of the same samples put in a water soak at 100 °C for 10 min. EC expresses the electrical conductivity

#### 4.3.11. Seed Yield and Related-Traits

At harvest, for the measurement of pods number plant^−1^, 100-seed weight and seed yield it was collected the aboveground tissues of soybean plants. The harvested plants were detached their pods to compute the numer of pods. Soybean seeds were measured kg ha^−1^.

#### 4.3.12. Seed Nutritional Value

For analysis of nitrogen, phosphorus, potassium concentrations (% seeds), 1 g soybean seed was milled and put with a 10 mL solution of mixed concentrated HClO_4_ and HNO_3_ acids (4:9 ratio) in flasks and boiled on a hot plate for 240 min. till colorless residue was set. Then used macro-kjldahle technique according toa [85] for nitrogen content and flame photometer according to [86] for phosphorus and potassium.

#### 4.3.13. Statistical Analysis

It was carried out by Origin Lab software version 8.4. The process started with the computation of descriptive statistics, including averages and standard deviations for each parameter. Following this, ANOVA was conducted, and Tukey’s HSD test was applied for post hoc comparisons to identify significant differences. To evaluate the relationships between parameter pairs across different treatments, Pearson correlation coefficients were calculated. Finally, PCA was used to decline dimensionality and assess the principal components that explain the dataset’s variance.

## 5. Conclusions

This study provides strong evidence for a sustainable, cost-effective, and eco-friendly approach to mitigate cadmium (Cd) and sodium (Na) ions in soybean cultivation on Cd-polluted saline soils irrigated with wastewater. The trio-combination of biochar, plant growth-promoting rhizobacteria (PGPR), and silicon nanoparticles (Si-NPs) consistently showed the most pronounced improvements in soil health, plant growth, and seed nutritional value. The combined treatments significantly reduced Cd and Na accumulation in plant tissues, with a 61.1% reduction in Cd in seeds and a 49% reduction in Na in leaves, as well as a notable increase in potassium (K) content by 267.31%. Furthermore, the trio-combination resulted in the highest enhancements in soil enzyme activities, with urease increasing by 166.92%, dehydrogenase by 229.04%, and alkaline phosphatase by 204.83%. The study also demonstrated a 94.68% increase in soil microbial biomass carbon, reflecting enhanced microbial activity and soil quality. This integrated approach improved antioxidant enzyme activity, reducing oxidative stress by 59.32% (MDA) and 78.4% (H_2_O_2_), while enhancing photosynthetic efficiency, which improved net photosynthesis by 136.12%. These findings suggest that applying biochar, PGPR, and Si-NPs together could offer a robust solution for farmers facing Cd-polluted saline soils, such as those irrigated with Kitchener Drain wastewater in Egypt. The improvements in soil properties, plant growth, and seed quality highlight this method’s potential in phytoremediation and sustainable agricultural practices. Future research could explore the use of biochar-based metal oxide nanoparticles in conjunction with PGPR under various abiotic stresses, further expanding the potential for field applications.

## Figures and Tables

**Table 1:** Characterization of irrigation water of Kitchener drain in the Summer growing season 2023.

Character	
pH	7.28 ± 01
EC (dS m^−1^)	0.55 ± 0.02
SAR	1.46 ± 0.04
**mg L^−1^**	
Na^+^	2.03 ± 0.11
Cl^−^	4.01 ± 0.08
NH_4_^+^	1.71 ± 0.03
SO_4_^− −^	0.12 ± 0.01
Cd	0.093 ± 0.01
Pb	0.088 ± 0.05
Ni	0.167 ± 0.06

**Table 2:** Soil biochemical analysis in the summer season 2023.

Character	2023
pH	8.32 ± 0.02
EC (dS m^−1^)	5.21 ± 0.01
Soil organic matter (g kg^−1^)	10.91 ± 0.02
ESP (%)	18.29 ± 0.39
Soluble cations	(meq L^−1^)
Ca^++^	6.89 ± 0.83
Mg^++^	5.65 ± 1.22
Na^+^	26.64 ± 2.11
K^+^	0.34 ± 0.01
Soluble anions	(meq L^−1^)
CO_3_^− −^	nd
HCO_3_^−^	4.52 ± 0.38
Cl^−^	24.44 ± 1.22
SO_4_^− −^	15.22 ± 3.11
Available macronutrients	(mg kg^−1^)
N	9.69 ± 0.88
P	8.13 ± 1.22
K	355 ± 26.31
	mg kg^−1^
Total Cd	5.15 ± 0.09
Available Cd	0.95 ± 0.04
Total Pb	18.38 ± 1.29
Available Pb	6.11 ± 0.02
Total Ni	39.56 ± 2.14
Available Ni	9.08 ± 0.01

**Table 3:** Biochar’s physicochemical characteristics during the summer growth season 2023.

Nutrients	Units	Values
pH	-	7.60 ± 0.02
EC	dSm^−1^	0.70 ± 0.01
CaCO_3_	%	1.4 ± 0.03
Bulk density	g cm^−3^	0.20 ± 0.03
Specific surface area	m^2^ g^−1^	37.0 ± 2.13
WHC	%	35.01 ± 2.23
Moisture	%	11.4 ± 1.09
	mg kg^−1^	
N		27.05 ± 3.08
P		8.02 ± 0.0.11
K		14.33 ± 1.87
Cd		0.01 ± 0.001

## Data Availability

Data will be made available upon the request.
